# A neutral theory of genome evolution and the frequency distribution of genes

**DOI:** 10.1186/1471-2164-13-196

**Published:** 2012-05-21

**Authors:** Bart Haegeman, Joshua S Weitz

**Affiliations:** 1 INRIA Research Team MODEMIC, UMR MISTEA, 34060 Montpellier, France; 2 School of Biology, Georgia Institute of Technology, Atlanta, GA 30332, USA; 3 School of Physics, Georgia Institute of Technology, Atlanta, GA 30332, USA

**Keywords:** Bacteria, Neutral model, Pan-genome, Population genomics, Selection

## Abstract

**Background:**

The gene composition of bacteria of the same species can differ significantly between isolates. Variability in gene composition can be summarized in terms of gene frequency distributions, in which individual genes are ranked according to the frequency of genomes in which they appear. Empirical gene frequency distributions possess a U-shape, such that there are many rare genes, some genes of intermediate occurrence, and many common genes. It would seem that U-shaped gene frequency distributions can be used to infer the essentiality and/or importance of a gene to a species. Here, we ask: can U-shaped gene frequency distributions, instead, arise generically via neutral processes of genome evolution?

**Results:**

We introduce a neutral model of genome evolution which combines birth-death processes at the organismal level with gene uptake and loss at the genomic level. This model predicts that gene frequency distributions possess a characteristic U-shape even in the absence of selective forces driving genome and population structure. We compare the model predictions to empirical gene frequency distributions from 6 multiply sequenced species of bacterial pathogens. We fit the model with constant population size to data, matching U-shape distributions albeit without matching all quantitative features of the distribution. We find stronger model fits in the case where we consider exponentially growing populations. We also show that two alternative models which contain a "rigid" and "flexible" core component of genomes provide strong fits to gene frequency distributions.

**Conclusions:**

The analysis of neutral models of genome evolution suggests that U-shaped gene frequency distributions provide less information than previously suggested regarding gene essentiality. We discuss the need for additional theory and genomic level information to disentangle the roles of evolutionary mechanisms operating within and amongst individuals in driving the dynamics of gene distributions.

## Background

The gene content of genomes of closely related bacteria can differ significantly. For example, pair-wise comparisons of genome sequences from isolates of the same species often do not share a substantial fraction of their gene content [1-10]. When a large number of genomes within a species or closely related group of bacteria are sequenced, the gene content variability can be summarized as a gene frequency distribution: given *G *sequenced genomes, some genes are found in a fraction 1 ≤ *k *≤ *G *of all genomes. Empirically, such gene frequency distributions possess a characteristic U-shape, such that there are many genes which only appear in one genome, fewer genes which appear in 2 ≤ *k *≤ *G *- 1 genomes, and many genes which appear in all genomes. Genes within each of these three categories have been labeled accessory, character and core genes, respectively [11]. It is tempting to conflate gene frequency with relative essentiality, but is it valid? For example, is it necessarily true that a gene that appears in all genomes in a sample should be classified as a "core" gene? Could such a gene have become common through neutral processes that diminish variability, and occasionally, lead to fixation of types? Likewise, should a gene which appears in only one genome in a sample be considered as "accessory" to the function of that organism? Could such a gene have become rare via a neutral path toward extinction, or have been recently introduced to a lineage without significant effect on individual fitness?

Here, we argue that a suitable null model is necessary with which to deduce how much weight be given to gene frequency data as a means to generate hypotheses regarding essentiality. For example, a recently proposed neutral theory of biogeography and biodiversity has proved useful in clarifying when and how species abundance distributions in ecology can provide information about selective processes in complex communities [12-15]. Hence, in this manuscript we ask: is it possible to recapitulate findings of U-shaped gene frequency distributions in the absence of selective forces driving genomic and population composition? We answer this question in the affirmative by proposing a simple and analytically tractable neutral model of genome evolution that explicitly accounts for gene composition of genomes. In this model, genomes undergo birth-death processes in a neutral sense and also acquire and lose genes which we term "gene transfer". The model differs from most previous efforts to analyze genome evolution [16,17] by self-consistently treating the dynamics at two scales: population level drift and genomic level change (for an exception, see [18] whose model we address in the Results and Discussion). Analysis of the current model leads to the following major results.

First, we find that gene frequency distributions derived from this model possess a characteristic U-shape for a robust range of model parameters. Hence, we propose that prevalence of a gene does not necessarily imply its essentiality, and that gene frequency distributions may be more limited than previously acknowledged in generating inferences regarding essentiality. Second, we estimate the best fit parameters for a given empirical gene-frequency distribution of sequenced genomes and in so doing find a reasonable correspondence between our neutral model and data from six distinct bacterial species with sequenced genomes from multiple isolates. However, our model assuming constant population sizes predicts gene frequency distributions with systematically fewer rare genes than the empirical distributions. Hence, we show that assuming other types of population dynamics (such as exponentially growing populations) can change the model predictions in line with empirical data, providing a basis for investigating the role of population dynamics in shaping gene frequency distributions. Further, we extend the model to include a rigid and flexible core in the genome, and show that other assumptions about genome structure are consistent with gene frequency data. Finally, we show that a recently proposed gene diversity index - genomic fluidity [19] - is a natural parameter emerging in the neutral models of genome evolution described here. Whereas previously this parameter was entirely statistical in nature, we discuss here how genomic fluidity can be seen as a proxy for the relative importance of gene uptake in shaping the gene composition of genomes between species. In so doing, we discuss how other observations could be combined with gene frequency distributions to improve inferences regarding evolutionary mechanisms shaping genome composition.

## Results and discussion

### A neutral model of genome evolution combines birth-death events with gene transfer events

**Figure 1:**
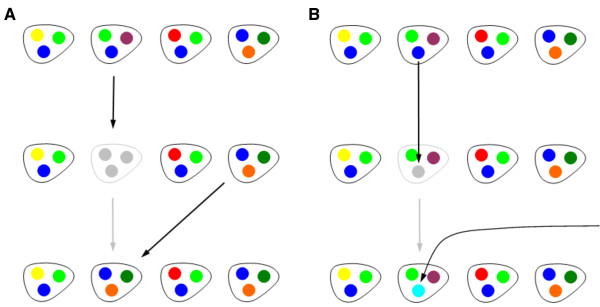
**Graphical illustration of a neutral model of genome evolution**. A population of *N *= 4 organisms is shown, with genomes consisting of *M *= 3 genes. Colors denote different gene identities. We chose small values for *N *and *M *for illustrative purposes; realistic values are, e.g., *N *~ 10^8 ^and *M *~ 2000. (A) In a birth-death event an organism dies and is replaced by offspring of another organism. The offspring genome is identical to the parent genome. (B) In a gene transfer event a gene of one of the organisms is replaced by a gene from the environment. We assume that this new gene has not been present in the population before

We propose the following neutral model of genome evolution, see Figure 1. Consider a population consisting of *N *organisms in which each organism has a genome consisting of *M *unique genes. The dynamics consist of a sequence of reproduction and gene transfer events. In a reproduction event, one of the *N *organisms (chosen at random) dies, and is replaced by offspring of one of the other organisms (chosen at random). The offspring genome is identical to the parent genome. Note that there are still *N *organisms after the event and hence, this step is equivalent to a birth-death event of the Moran model [20,21]. In a gene transfer event, one of the *N *organisms acquires a gene from the environment. We assume that this gene is new, i.e., a gene that has not been present in the population before, and hence, this step is comparable to a mutation event in the infinitely many alleles model of population genetics [20,21]. In the model, gene transfer events do not affect birth and death rates of the individual (e.g. [22] present evidence for the neutrality or near-neutrality of transferred genes). We also assume that the acquisition of the new gene induces the loss of another gene in the genome, so that the organism's genome still consists of *M *genes after the event. We utilize a constant value of *M *to facilitate mathematical analysis and note that bioinformatic based estimates of total gene counts vary approximately 10% between genomes of the same species, as considered here.

Individual birth-death events cause genetic drift in the population as the number of organisms having a particular gene fluctuates over time. Genetic drift has the tendency to reduce the genetic diversity in the population. Indeed, when the last organism carrying a particular gene dies, this gene disappears from the population, and has no opportunity of re-entering the population. Gene birth-death events, on the other hand, maintain the genetic diversity in the population. New genes enter at low frequency due to "gene transfer" events. These transfer events may be due to uptake of genes from the environment, insertion of genes via viruses, or conjugation with other individuals. In our model, individual birth-death events and gene transfer events have associated rate parameters: we denote by *ρ *the rate of reproduction per individual, and by *σ *the rate of gene transfer per individual. Intuitively we expect that variability in gene composition of genomes should increase when gene transfer rate *σ *increases relative to the individual reproduction rate *ρ*, and vice-versa. Further, we expect that gene frequency distributions will tend toward U-shaped distributions because of the tension between gene transfer (which would favor increasing rarity of genes) and individual birth-death (which would favor increasing commonness of genes, due to neutral drift). We evaluate this prediction of U-shaped distributions in the following section.

### Neutral model of genome evolution predicts U-shaped gene frequency distributions

The distribution of genes over genomes can be characterized in detail for the neutral model of genome evolution described above. For example, the gene frequency distribution can be computed explicitly, see Additional file 1: Appendix S1. To describe the solution, we consider a sample of *G *genomes taken from the population, and we denote the average number of genes appearing in *k *of the *G *genomes by *g*_*k*_. The gene frequencies predicted by the neutral model of genome evolution are

(1) $$g_{k}=\frac{M\theta }{k}\frac{G!}{\left(G-k\right)!}\frac{{\left(\theta \right)}_{G-k}}{{\left(\theta \right)}_{G}},$$

with

$$\theta =\frac{N\sigma }{M\rho }\;\text{and}\;{\left(\theta \right)}_{k}=\theta \left(\theta +1\right)\ldots \left(\theta +k-1\right),$$

where *θ *is an effective gene transfer rate. The distribution in Eq.(1) appears in solutions to allele distributions in the infinitely many alleles model of population genetics [20,21].

As the number of genes *M *in a genome and the number of genomes *G *in the sample are given by the data, the gene frequencies *g*_*k *_are parametrized by the dimensionless parameter *θ *which combines the effects of both gene transfer and birth-death processes. Hence, different combinations of *N*, *ρ *and *σ *lead to identical gene frequency distributions. In particular, the predicted distribution is insensitive to accelerating simultaneously gene transfer and reproduction, because the distribution depends on the ratio $\frac{\sigma }{\rho }$, and not on *ρ *and *σ *individually. Moreover, an increase of the ratio $\frac{\sigma }{\rho }$, the relative rate of gene transfer, can be compensated by a smaller population size *N*, increasing the intensity of genetic drift. Note that although in practice the sample size *G *is much smaller than the population size *N*, Eq. (1) is also valid for *G *= *N*, i.e., it can be used to compute the (empirically inaccessible) gene frequency distribution of the entire population.

**Figure 2:**
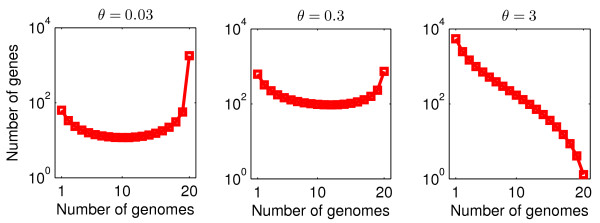
**Gene frequency distributions for neutral model of genome evolution (model A)**. Genome size *M *= 2000 and sample size *G *= 20. Gene transfer parameter *θ*: in left panel, *θ *= 0.03; in middle panel, *θ *= 0.3; in right panel: *θ *= 3

We plot Eq. (1) for cases where *θ *= 0.03, *θ = *0.3 and *θ = *3 in Figure 2. As anticipated, the weight of the gene frequency distribution shifts from the common genes for small values of *θ *(left panel) to the rare genes for larger values of *θ *(right panel). The gene frequency distributions have a U-shape for a robust range of parameters. The U-shape is generic so long as *θ <*1; for *θ >*1 the distribution is monotonically decreasing. As shown in Additional file 1: Figure S1, the gene frequency distribution changes when sampling more genomes, but the characteristic U-shape remains. These observations for the neutral model of genome evolution show that U-shaped frequency distributions do not require invoking selection at the genome level. Further, the observations suggest that findings of prevalent genes need not be an indicator of essentiality in the absence of other information about gene function.

### Comparing empirical gene frequency distributions of multiply sequenced bacterial species to model predictions

**Figure 3:**
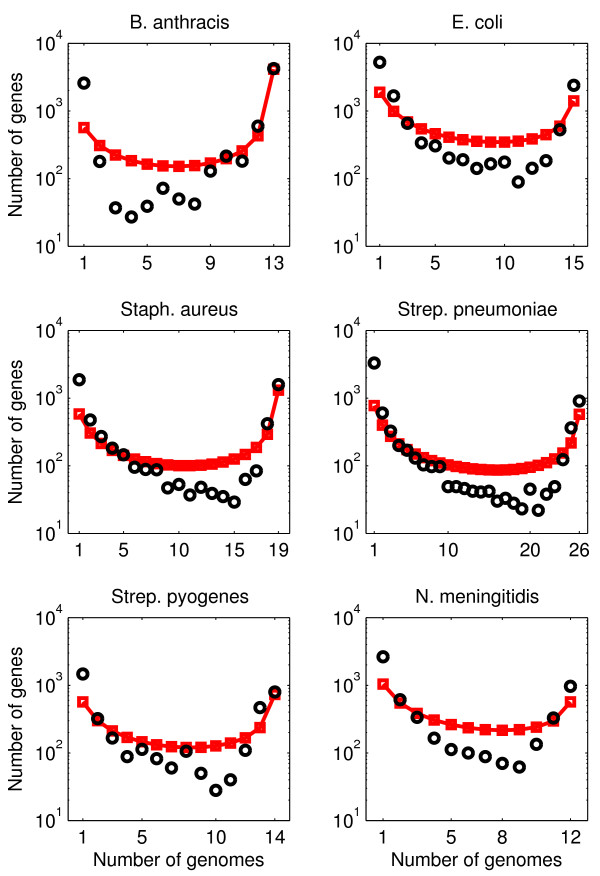
**Data comparison for neutral model of genome evolution (model A)**. Comparison of gene frequency distributions with predictions of the simplest model: the population size is assumed to be constant and all genes are governed by the same gene transfer process. The model has one parameter, the gene transfer parameter *θ*. Black circles: data; red line with squares: model

We collect and analyze empirical gene frequency distributions from 6 species of bacterial pathogens: *B. anthracis, E. coli, Staph. aureus, Strep. pneumoniae, Strep. pyogenes *and *N. meningitidis*. Gene frequency distributions were compiled by applying an automated genomic pipeline to remove the impact of curation bias and to normalize comparisons between species [23]. Hence, what we compile are frequency distributions of clusters of homologous genes (for details, see [19,23]), which we denote, for simplicity, as "genes" in this manuscript. We find that the empirical gene frequency distributions have a characteristic U-shape in that there are many genes which only appear in a single genome, many genes which appear in all genomes, and fewer genes that appear in an intermediate number of genomes (see Figure 3). This characteristic U-shape is robust to reasonable changes in the values of identity and coverage utilized for comparing genes in our genomic pipeline. Notice that the gene frequency distribution is on a log scale, hence the U-shape is in fact highly pronounced, in that there may be 50 times as many genes that appear in all genomes than appear in half the genomes.

The neutral model of genome evolution can be fit to data using Eq. (1). To do so, we determine the parameter *θ *that minimizes the distance between the predicted and empirical gene frequency distribution (see Materials and Methods). We find that the neutral model is in reasonable correspondence with data (see Figure 3). First, both data and predictions have a U-shape. Next, model predictions agree well with observations for the total number of core genes. These predictions are made with a single free parameter, *θ*. On the other hand, the model underpredicts the number of rare genes and overpredicts the number of genes in the intermediate part of the distribution. In particular, the model predicts a *g*_*k *_~ 1*/k *dependence for the peak at small *k*, whereas the data are closer to a steeper *g*_*k *_~ 1*/k*^2 ^dependence. In the next section we show that this deviation can be partially remedied by dropping the assumption of a constant population size. Finally, it is important to note that although our model assumes constant *M*, we find that there is approximately a 10% difference in total gene content within the genomes of each of the six species.

Predictions for the observed pan and core genome size in a sample of genomes can also be obtained. In the past, the pan genome size has been defined as the number of genes in all genomes of the population. Similarly, the core genome size has been defined as the number of genes found in every genome in the population. However, we and colleagues have previously shown that estimating pan and core genome sizes are unreliable because they depend on observations of rare genes and genomes, respectively, which are difficult to find in samples precisely because they are rare [19]. Here, we define the observed pan and core genome size as the number of unique genes found in all sample genomes and the number of genes common to all sample genomes, respectively. As we derive in Additional file 1: Appendix S1, the model predicts that the pan genome size increases logarithmically with sample size, and that the core genome size decreases as a power law (with exponent -*θ*). The prediction that gene diversity grows without bound is unsurprising, because we assumed an infinite gene pool (and we caution that gene diversity cannot increase to infinity in reality). Nevertheless, we expect the logarithmic (power-law) dependence of pan (core) genome size also to hold for a finite gene pool (as long as the gene pool is much larger than the set of genes observed in the sample). These findings are corroborated by the data, which exhibit the same qualitative behavior, see Additional file 1: Figure S2. Notice that we used the value for *σ *obtained from Figure 3 in the pan and core genome fits of Additional file 1: Figure S2, so that these fits have no additional free parameters.

### Estimating gene transfer parameters from model fits

We can utilize parameters estimated from gene frequency distribution fits to also estimate underlying mechanistic parameters driving genome evolution, albeit with caveats that we discuss below. Note that the estimated values for *θ *are in the range 0.1 <*θ *< 0.5 (see Table 1). For example, with *M *≈ 2000, then the product *Nσ*/*ρ *should be on the order of 10^3^. Conventional estimates of effective population size are approximately 10^7 ^- 10^9 ^[24,25], suggesting that gene uptake is on the order of *σ*/*ρ *≈ 10^-4 ^- 10^-6^, approximately once per tens of thousands or million divisions. Assuming bacteria that divide once per hour, then *σ *≈ 10^-4 ^- 10^-6 ^hr^-1^. In this model, *σ *represents gene transfer. Here, we consider one mechanism for such gene uptake - natural uptake of DNA from the environment - and evaluate whether or not empirical parameters associated with transformation are consistent with inferred estimates of *σ*. Natural transformation rates vary widely depending on strain type, sequence homology, and physiological conditions. Empirically estimated DNA uptake rates are generally reported as transformation frequencies, *ϵ*, defined as the proportion of colony forming units (CFUs) that have taken up a segment of DNA of interest at the end of some experimental time period, *T*. We developed a simple model that estimates *σ *directly from *ϵ *and *T *(see Materials and Methods). We find that application of this method yields estimates of gene uptake rate for pathogens in laboratory environments that bracket the value predicted from our model. For example, DNA damaged *Helicobacter pylori *cells exhibit *ϵ *~ 10^-4 ^- 10^-8 ^in a *T *= 2.5 hr experiment [26] Hence, *σ *~ 10^-4 ^- 10^-9 ^hr^-1^. Likewise, naturally competent *Neisseria gonorrhoeae *cells exhibit transformation frequencies *ϵ *~ 10^-3 ^of total cells after *T *= 4 hr, though values range from *ϵ *~ 10^-2 ^- 10^-7 ^[27]. These values yield uptake rates of *σ ~ *10^-4 ^hr^-1 ^with a range of *σ *~ 10^-3 ^- 10^-8 ^hr^-1^. In both cases, these estimates are consistent with our estimate of gene transfer rates in the multiply sequenced pathogens considered here. However, there remains substantial disagreement as to whether the lower, effective number derived from certain population genetic models or the much larger, census number is a better estimate of effective population size [25]. Hence, without species-specific information, we caution that direct estimates of either gene transfer rate or effective population size should be treated with skepticism, even if estimates of their combined effect is more robust. Moreover, as we show in the next section, the value of *θ *is sensitive to assumptions about population history. Indeed, there is no reason to expect that the population structure and selective effects of genes are as simple as assumed here, providing additional caution to overly rigid interpretations of estimates of either *N *or *σ*.

**Table 1:** Overview of model fits

				fitting error					fluidity		
	***G***	***M***	**Δ_A_**	**Δ_B_**	**Δ_C_**	**Δ_D_**	***φ*^obs^**	$\varphi_{\text{A}}^{\text{pred}}$	$\varphi_{\text{B}}^{\text{pred}}$	$\varphi_{\text{C}}^{\text{pred}}$	$\varphi_{\text{D}}^{\text{pred}}$

*B. anthracis*	13	5523	80	21	78	13	0.08	0.09	0.08	0.09	0.08
*E. coli*	15	4576	98	58	47	2.6	0.25	0.30	0.25	0.29	0.25
*Staph. aureus*	19	2651	29	16	21	4.3	0.16	0.19	0.16	0.19	0.16
*Strep. pneumonia*	26	2095	42	21	30	4.3	0.23	0.32	0.24	0.30	0.23
*Strep. pyogenes*	14	1786	26	10	25	7.5	0.20	0.24	0.20	0.24	0.21
*N. meningitidis*	12	2080	53	26	31	2.4	0.28	0.33	0.28	0.32	0.28

Model A assumes a constant population size, and the same gene transfer process for all genes. Model B assumes an exponentially growing population size. Model C assumes that a part of the genome is shared by all genomes (a rigid core); the other part is subjected to the same gene transfer process as in model A. Model D assumes two parts in the genomes, governed by different gene transfer rates. We determined for the four models the parameters that minimize the distance Δ between the empirical and the theoretical gene frequency distribution (see Materials and Methods for the definition of Δ). For each of the 6 bacterial species analyzed, we report the number of analyzed genomes *G*, the genome size *M *(average number of genes per genome), the distance Δ for the model fits, the genomic fluidity *φ*^obs ^estimated on the data, and the fluidity *φ*^pred ^for the model fits. Recall that model A has one parameter, models B and C have two parameters, and model D has three parameters.

### Population structure strongly impacts gene frequency distributions

The current neutral model of genome evolution assumes a fixed population size *N*. This is a common, but likely unrealistic, assumption as bacterial populations can undergo large and fast size fluctuations. In this model, the introduction of novel genes is decoupled from the history of population size or structure, so that we can select an arbitrary population size or structure and then superimpose the introduction of novel genes on top of the resulting history of individual births and deaths. To illustrate this point, we consider how an exponentially growing population affects the gene frequency distributions *g*_*k*_. Specifically we denote the population size history as

(2) $$N\left(t\right)=N_{0}{\text{e}}^{\alpha \left(t-t_{0}\right)},$$

with *α *the population growth rate, *t*_0 _the present time and *N*_0 _= *N*(*t*_0_) the present population size. We use a coalescence approach [20,21] to compute the average gene frequencies *g*_*k*_, see Additional file 1: Appendix S3. The solution for the average gene frequency distribution *g*_*k *_depends on two dimensionless parameters *θ*_0 _and *β*,

(3) $$\theta_{0}=\frac{N_{0}\sigma }{M\rho }\;\text{and}\;\beta =\frac{N_{0}\alpha }{2\rho }.$$

The parameter *θ*_0 _is the same as *θ *for the constant population size model, except that the population size *N *is replaced by the present population size *N*_0_; again we call *θ*_0 _the gene transfer parameter. The parameter *β *is a rescaled version of the population growth rate *α*; we call it the population growth parameter. The constant population size model, which we denote by model A, is a one-parameter model; the variable population size model, which we denote by model B, is a two-parameter model. Hence, we expect a richer set of gene frequency distributions predicted by model B compared to model A.

Additional file 1: Figure S3 shows gene frequency distributions computed for different combinations of the parameters *θ*_0 _and *β*. For small *β *≤ 1 the distributions closely resemble the distributions of the model A with constant population size (*α *= *β *= 0, see Figure 2). When increasing the population growth parameter *β*, the U-shape becomes more pronounced. For example, the peak at small *k *has a power-law dependence *g*_*k *_~ *k*^*-γ *^with *γ = *1 for small *β *and *γ *increasing for increasing *β*. The predicted distributions are often, apart from the peak for the core genes present in all genomes, almost symmetric (see panels with *θ*_0 _= 0.03 or *θ*_0 _= 0.3). Notice that very similar distributions can be obtained for different parameter combinations (e.g., compare panel *θ*_0 _*= *0.03, *β *= 10 and *θ*_0 _= 0.3, *β *= 100), which will affect the parameter estimation (see below).

**Figure 4:**
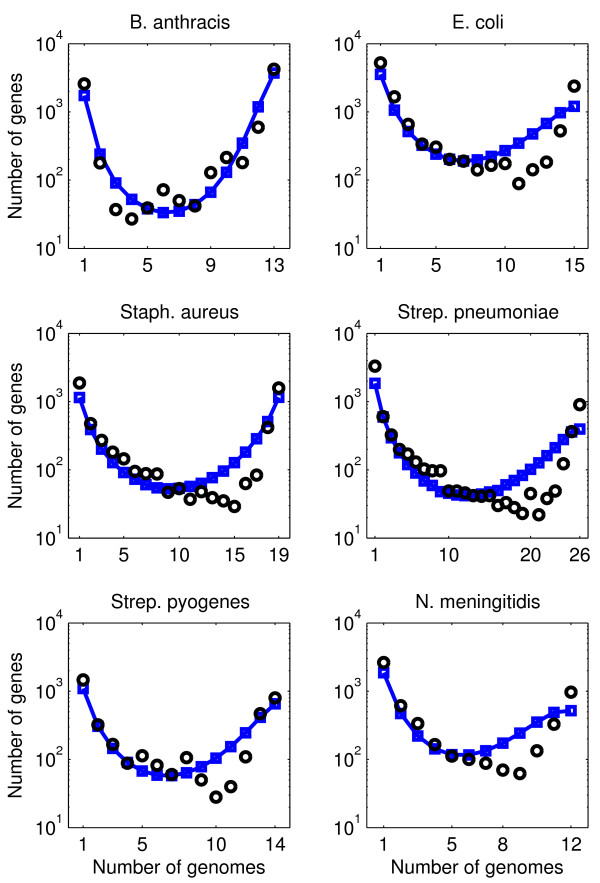
**Data comparison for model with exponentially growing populations (model B)**. Comparison of gene frequency distributions with predictions of the model in which population size is assumed to grow exponentially. The model has two parameters, the gene transfer parameter *θ*_0 _and the population growth parameter *β*. Black circles: data; blue line with squares: model.

We can fit empirical distributions to this neutral model of genome evolution (model B). To do so, we determine the parameters *θ*_0 _and *β *that minimize the distance between the predicted and empirical gene frequency distribution. This computation yields estimates for the gene transfer parameter *θ*_0 _and the population growth parameter *β*. As shown in Figure 4, the gene frequency distributions for model B fit the data better than those for model A (compare with Figure 3). The predictions of model B are uniformly accurate for the number of rare genes, the number of genes in the intermediate part of the distribution and the number of common genes. However, smaller but systematic deviations remain between observed and predicted gene frequency distributions. In particular, the empirical distributions (except for *B. anthracis*) are left-skewed, whereas the theoretical distributions have no skew or a small right skew. The improved fit is also apparent from the distance Δ between data and model, reported in Table 1, especially for *B. anthracis*. The estimate of the gene transfer parameter *θ*_0 _is an order of magnitude larger in model B than the estimate of the gene transfer parameter *θ *in model A. However, the population size in model B is that of the present, whereas the population size in model A is the effective population size over the entire coalescent history. Hence, differences in gene transfer parameters are to be expected because they are driven in part by changes in assumptions about population size. This suggests that caution must be applied before utilizing *θ*, *θ*_0_, or other dimensionless gene transfer parameters, to estimate an effective gene transfer rate without additional information that constrains the estimates of both population size and growth rate. For similar reasons caution should be applied to the interpretation of the estimates of the growth parameter *β*.

### Models with an explicit core genome improve fit of empirical gene frequency distributions

The previous models assume that the gene transfer process affects all genes identically. Indeed, each gene present in a genome has the same chance to be replaced by a gene transfer event, and this replacement has no effect on the reproduction rate. Here we show how to relax this assumption without prohibitively increasing the model complexity. To illustrate this, we study a new model, in which we distinguish two parts in the genomes: one part is governed by the same gene transfer process as model A; the other part does not undergo gene transfer and hence, constitutes a rigid core genome. We term this model C and note that a similar model has also been proposed in the context of the analysis of *Prochlorococcus *genomes [18]. We assume that this rigid core has the same composition for all genomes. One interpretation of the rigid core is that genes in this core are essential, and deletion of any of a subset of genes in the core would be lethal to the individual. The average gene frequency distribution is given by Eq. (1), with an additional contribution for the rigid core, see Additional file 1: Appendix S5. This distribution depends on two parameters: the fraction *λ*_1 _of the fluid part of the genomes, corresponding to *λ*_1_*M *genes per genome, and the gene transfer parameter *θ*_1 _for this part. The rigid core then represents a fraction *λ*_2 _***= ***1 - *λ*_1_, or *λ*_2_*M *genes.

**Figure 5:**
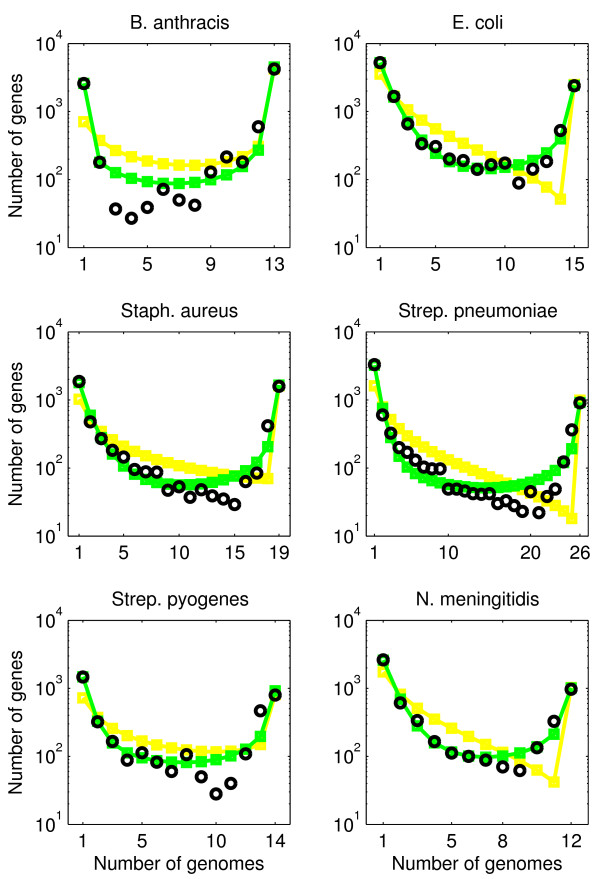
**Data comparison for models with rigid and flexible core genomes (models C and D)**. Comparison of gene frequency distributions with predictions of two models which assume that a part of a genome is more susceptible to gene transfer. The genomes in model C have a rigid core, i.e., some genes cannot be removed from the genomes. The genomes in model D have a flexible core, i.e., theses core genes can be moved around between genomes, but to a lesser extent than the other genes. Model C has two parameters, whereas model D has three parameters. Black circles: data; yellow line with squares: model C; green line with squares: model D.

We can determine the parameters for which model C fits best the empirical gene frequency distributions. The model fits, shown in Figure 5 (yellow line), are better than those for model A (Figure 3), but are worse than those for model B (Figure 4), see also Table 1. Note that the fitting error Δ_*B *_and Δ_*C *_of models B and C can be compared directly, because both models have two independent parameters. Model C predicts that about half of the genome belongs to the rigid core (*λ*_1 _≈ *λ*_2 _≈ 0.5, see Table 2). The other part of the genome is rather fluid, with estimated gene transfer parameter *θ*_1 _≈ 1 (see Table 2). This combination of parameters results (except for *B. anthracis *and *Strep. pyogenes) *in a steep dip for the common (but not core) genes of the frequency distributions. However, such a dip is not present in the data (Figure 5).

**Table 2:** Parameter values of model fits

	model A	model B		model C		model D	
	***θ***	***θ*_0_**	***β***	***θ*_1 _(*λ*_1_)**	***θ*_2 _(*λ*_2_)**	***θ*_1 _(*λ*_1_)**	***θ*_2 _(*λ*_2_)**

*B. anthracis*	0.10	6.9	490	0.30 (0.41)	0 (0.59)	∞ (0.03)	0.06 (0.97)
*E. coli*	0.44	2.1	17	1.77 (0.46)	0 (0.54)	12 (0.15)	0.15 (0.85)
*Staph. aureus*	0.24	0.87	10	0.92 (0.42)	0 (0.58)	14 (0.07)	0.12 (0.93)
*Strep. pneumonia*	0.48	1.94	17	1.47 (0.53)	0 (0.47)	41 (0.08)	0.20 (0.92)
*Strep. pyogenes*	0.33	1.93	23	0.57 (0.68)	0 (0.32)	40 (0.06)	0.20 (0.94)
*N. meningitidis*	0.50	3.5	30	1.72 (0.52)	0 (0.48)	15 (0.16)	0.19 (0.84)

We determined for each of the four models A, B, C and D the parameters that minimize the distance Δ, see Eq. (6), between the empirical and the theoretical gene frequency distribution. Here we report the gene transfer parameter *θ *of the model A fit, the gene transfer parameter *θ*_0 _and the population growth parameter *β *of the model B fit, the genome fractions *λ*_1 _and *λ*_2_, and the gene transfer parameter *θ*_1 _of the model C fit, and the genome fractions *λ*_1 _and *λ*_2_, and the gene transfer parameters *θ*_1 _and *θ*_2 _of the model D fit.

**Figure 6:**
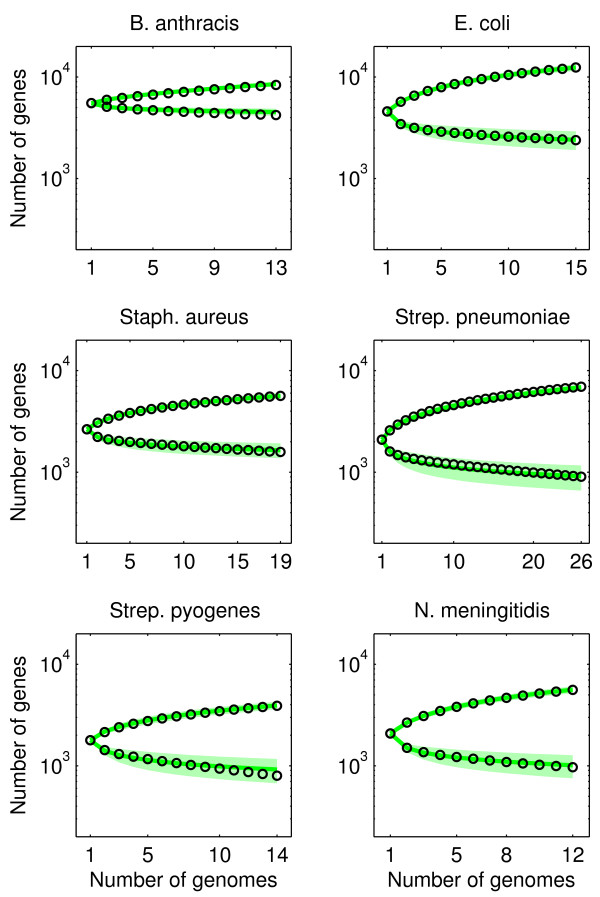
**Predictions for observed core and pan genome size for model D**. We used the parameters λ_1_, *θ*_1 _and *θ*_2 _obtained from fitting the gene frequency distribution (see Figure 5) to evaluate the predicted core and pan genome size (see Additional file 1: Appendix S6). Black circles: data; green line: mean prediction; green shaded region: standard deviation of prediction. The increasing curves are for the pan genome; the decreasing curves are for the core genome.

To weaken the assumption of a rigid core, we consider another model with an explicit core genome, which we call model D, in which genes in the core genome retain some fluidity. More precisely, as for model C, we divide the genomes into two parts. Both parts are governed by the gene transfer process of model A, but the genes in the first part (fraction *λ*_1_, gene transfer parameter *θ*_1_) are more fluid than the genes in the second part (fraction *λ*_2 _= 1 - *λ*_1_, gene transfer parameter *θ*_2 _<*θ*_1_). Hence, model D has three independent parameters. The average gene frequency distribution is equal to the sum of two distributions (1), with parameters *θ*_1 _and *θ*_2_, see Additional file 1: Appendix S5. The predicted distributions show an excellent agreement with the empirical data (Figure 5), as can also be seen from the fitting error Δ: for *E. coli *and *N. meningitidis *the error has dropped tenfold compared to the other models, see Table 1. It is also interesting to note that the model consistently (for the 6 bacterial species, although *B. anthracis *clearly stands out) predicts genomes with a small part of high fluidity (*λ*_1 _≈ 0.1, *θ*_1 _≈ 10) and a large part of low fluidity (*λ*_2 _≈ 0.9, *θ*_2 _≈ 0.1). Moreover, the model with a flexible core genome (model D) predicts the scaling of sample pan and core genome sizes in close agreement with the data, see Figure 6. Further, because no additional free parameters were utilized in making this fit, such scaling represents an additional prediction of each of the models.

### Genomic fluidity as a mechanistic summary statistic for gene frequency distributions

In a previous work [19] we advocated for the use of robust diversity indices to describe gene variation between genomes. In doing so we proposed the use of "genomic fluidity" which captures the average dissimilarity of pairs of genomes from within a group based on gene content. Specifically, genomic fluidity is equal to the probability that a randomly chosen gene from one genome is not found in another genome within the same group of organisms. For a sample of *G *genomes it can be estimated using the following formula:

(4) $$\varphi =\frac{2}{G(G-1)}\sum_{\underset{k<\ell }{k,\ell =1}}^{G}\frac{U_{k}+U_{\ell }}{M_{k}+M_{\ell }},$$

where *U*_*k *_and *U*_ℓ _are the number of genes found in either (but not both) genomes *k *and ℓ respectively in a pairwise comparison, and *M*_*k *_and *M*_ℓ _are the total number of genes found in genomes *k *and ℓ respectively. Estimates of genomic fluidity within a sample should agree with the true value of genomic fluidity within the population, in part, because they do not depend on the frequency of rare genomes or genes [19]. Note that genomic fluidity summarizes gene frequency distributions, however multiple gene frequency distributions may be compatible with the same value of genomic fluidity. Hence, here we ask whether genomic fluidity is related to the model parameters, *θ*, *θ*_0_, and *β*, that underlie the gene frequency distributions presented here.

For the model with constant population size, the genomic fluidity *φ *and the gene transfer parameter *θ *are intimately linked. For a population in steady state, we have, see Additional file 1: Appendix S1,

(5) $$\varphi =\frac{\theta }{1+\theta }\;\text{or}\;\theta =\frac{\varphi }{1-\varphi }.$$

Hence, genomic fluidity has a one-to-one relationship with *θ*, the relative rate of gene uptake to genomic replacement. When genomic fluidity approaches 1, then genomes are nearly completely dissimilar, which implies large gene replacements relative to genome reproductions (large *θ*). When genomic fluidity approaches 0, then processes that promote convergence of genomes are more important than gene-uptake processes (small *θ*). Previously, we advocated for the use of genomic fluidity on statistical grounds as a means to compare gene diversity between groups of genomes and as an alternative to the estimation of pan and core genome diversity. The constant population size model demonstrates that genomic fluidity may be indicative of processes driving the uptake of genes from the environment vs. genetic drift.

For the model with exponentially growing population size, the relationship between the genomic fluidity *φ *and the parameters *θ*_0 _(for gene transfer) and *β *(for population growth) is more intricate. Genomic fluidity increases with the gene transfer parameter *θ*_0 _and with the population growth parameter *β*, but there is no simple formula for *φ*(*θ*_0_, *β*) analogous to Eq. (5). However, the genomic fluidity is useful to clarify the estimation of the parameters *θ*_0 _and *β*, see Additional file 1: Appendix S4. Indeed, the different model fits return very similar estimates for the genomic fluidity (including the models with rigid and flexible cores), see Table 1. This illustrates the robustness of genomic fluidity, confirming our previous findings [19]. However, this robustness comes with a trade-off: because very different parameter combinations *θ*_0 _and *β *have the same genomic fluidity, we are unable to infer the gene transfer parameter *θ*_0 _and the gene transfer rate *σ *from the genomic fluidity alone. This is a very typical finding in dynamic models in that predictions can be robust even when inferences of exact combinations of mechanistic parameters may not always be possible from model fits [28].

## Conclusions

We have presented a neutral model of genome evolution that combines birth-death processes at the population level with gene transfer events at the genome level. We find that this model generically yields U-shaped gene frequency distributions. This result suggests that a gene's prevalence is insufficient to infer its essentiality to a species. We compared our model to empirical gene frequency distributions estimated from sequenced genomes of six bacterial species and found: (i) reasonable fits to data; (ii) improved fits when assuming non-constant population size or including an explicit core genome; (iii) despite the qualitative agreement, that there still remains unexplained aspects of empirical gene frequency distributions, e.g., skewness; (iv) that our neutral model is remarkably compatible with a previous proposal for a robust gene diversity index - genomic fluidity [19]. We have also shown that our modelling framework can easily incorporate more complexity, which not surprisingly gives improved fits. In this sense our model is also formally related to models of population genetics in which assumptions of population sizes and dynamics are meant to evaluate if and when spatial population structure and even ecological dynamics may alter allele distributions in identifiable ways [20,21]. In the present case the excellent fits obtained, e.g., for our flexible core model (model D), should not be interpreted as an indication for the validity of its assumptions. Rather, our analysis shows that gene frequency distributions do not contain sufficient information for the inference of evolutionary mechanisms underlying the observed distributions. Moreover, the finding that neutral models can generically lead to U-shaped gene frequency distributions suggests the need to incorporate and evaluate random processes in the analysis of gene composition and its dynamics.

Horizontal gene transfer is widely recognized as being an important mechanism driving genome evolution [22,25,29-31]. As such, there are many other models of evolution that address how neutral and selective processes give rise to variation in the state of genes and genomes (e.g., [16,17,20,32-35]). Indeed, the central model of population genetics in which individuals die and are replaced at random by other individuals is utilized here [20,21]. However, in the current model, genetic variation arises via the uptake of a novel gene. A recent paper also proposed a model of genome dynamics in which a rigid core was imposed [18] in order to fit gene frequency distributions estimated from 9 *Prochlorococcus *genomes. As shown here, such a fit may have limited inferential value, since a rigid core is not necessary in order to model gene frequency distributions. However, prior modeling suggests multiple avenues by which our model can be unified with dynamics at different scales. First, we have not considered horizontal gene transfer within genomes of the same species, nor recombination during division, nor of other types of transduction that may help to explain the finer genetic structure of bacterial populations [35-37]. Note that within-species gene transfer makes the acceptor genome more similar to the donor genome, and therefore reduces genetic diversity in the population just like genetic drift. We expect within-species gene transfer to have a smaller impact on genetic diversity than birth-death events, although its quantitative effect on the gene frequency distribution might be different (see [38] for an attempt to account for within-species gene transfer in a model similar to ours). Second, we do not include the fitness effect of mutations, whether neutral, beneficial or deleterious, which would impact the fixation of novel as well as pre-existing genes in genomes [16,17]. Including non-neutral mutational effects would obviously be a departure from our effort here to describe how much of the information on gene variation in genomes can be described using purely neutral models or simple extensions thereof. Note that although the impact of horizontally transferred genes on genome fitness remains controversial, there is evidence that such genes have no, or mild, effects on genome fitness [22]. Finally, a number of models have taken steps toward describing how the sizes of groups of genes, protein domains, proteins, and even categories of proteins (e.g., transcription factors) have changed over long evolutionary scales [32-34,39,40]. These models typically describe the structure within a genome (e.g., the abundance distribution of protein domains within different domain classes [34]), whereas our model describes population structure. It would seem that some unification of these models may be possible.

Development of models to predict and characterize gene composition variation among genomes is motivated by improvements in sequencing technologies which have enabled whole-genome sequencing of multiple isolates of the same bacterial species [41]. However, the gene frequency distribution data upon which we base this model is subject to two caveats. First, we treated the sequenced genomes as if they were sampled uniformly from the population. However, the genomes exhibit phylogenetic structure which should be taken into account. One option would be to use the total divergence of the core genes to correct for the non-uniform sampling (e.g., [42]), although alternative normalizations are possible. Second, determining whether two genes are found in a pair of genomes depends on the use of cutoffs within some comparative alignment scheme. Different cutoffs can be utilized depending on whether one is interested in gene homologs, orthologs, gene families, gene super-families, and so on. If the cutoffs are set too stringently, then nearly every gene will appear to be unique. If the cutoffs are set too loosely, then every gene will appear to be the same as every other. Prior work demonstrated that there exist metrics of gene composition dissimilarity (e.g. genomic fluidity) that are robust to changes in such cutoffs [19]. A unification of the current model with a sequence-based gene model would present opportunities to connect more factors (including mutation and recombination) driving gene variation with empirical patterns. However, we suggest that caution may be necessary in moving forward when attempting to utilize best fit parameters to infer mechanistic rates. In the present case, we showed that our neutral model reveals a well-known phenomenon of having robust predictions within a parameter space that poses an identifiability problem [28]. In essence, there are combinations of evolution parameters that yield similar predictions for gene frequency distributions (see Additional file 1: Figure S5 and Additional file 1: Appendix S4). Hence, more information is required concerning actual population size structure and the nature of gene uptake [43] before we recommend utilizing our best fits to precisely estimate gene transfer rate, effective population size, growth rate and so on. More generally, fitted parameter values are subject to numerous simplifying assumptions of the models. Although it is interesting to compare the order of magnitude of the parameter fits with experimental data, one should be cautious to interpret the parameters too strictly as directly measurable quantities.

In this manuscript we presented a purely neutral explanation for the non-equal distributions of genes within genomes. The utilization of neutral models in genetics and ecology have yielded similar results in the past: in presenting quantitative arguments for when unequal patterns of appearance imply mechanisms of selection [12,44,45]. For example, a recent proposal for a unified theory of biodiversity and biogeography for forest trees [12] started with a similar dilemma. In that case, ecologists had observed skewed rank-abundance relationships such that some tree species were found at very high abundances and others at very low abundances. Ecologists had by and large assumed that those trees with greater abundance had a fitness advantage over trees with lower abundances. However, Hubbell's model showed that finding a few common trees and many rare trees could also be derived without invoking selection. Hence, in order to determine whether or not tree species had a fitness advantage in different regions one needed to look for correlations between traits and abundance which would not have been expected from a purely neutral model [46,47]. In the case considered here, our neutral model shows that the U-shape of gene frequency distributions provide less information than previously thought about the fitness benefit of genes. Instead, we need to find patterns of genome composition variation that can be explained by neutral models and identify those patterns or deviations from patterns that cannot be explained by neutral models - similar proposals have been advocated in other contexts [48]. Possible examples include the analysis of gene sequences and correlations amongst those gene present or absent amongst a set of genomes. In moving forward we suggest the need to continue to build the toolbox of a quantitative evolutionary genomics specifically adapted to the mechanisms operating within and amongst microbes.

## Methods

### Empirical estimation of gene frequency distributions

The pipeline has been described in detail elsewhere [19,23]. In brief, it (i) finds genes; (ii) calculates homology between all genes within a group of genomes using a set of cutoffs associated with identity and coverage (here set at 70% identity and coverage); (iii) applies a maximal clustering rule to determines groups of homologous genes; (iv) determines a gene presence-absence matrix of dimension *M*_tot _× *G *of the total number of genes *M*_tot _in the group of *G *genomes. We take row-sums of this matrix to find the frequency of each of the *M*_tot _genes, and then take the histogram of these row-sums to calculate the gene frequency distribution. See Additional file 2 for the empirical gene frequency distributions of each of the six species analyzed here.

### Estimating model parameters given empirical data

The parameter estimation is based on the average gene frequencies *g*_*k*_. For model A the frequencies are computed using Eq. (1). For model B the frequencies are computed using the algorithm of Additional file 1: Appendix S3. For model C and D the frequencies are computed using the equations of Additional file 1: Appendix S5. To fit an empirical gene frequency distribution, we determine the model parameters that minimize the distance between the observed distribution $g_{k}^{\text{obs}}$ and the predicted distribution $g_{k}^{\text{pred}}$. We use the following distance function Δ,

(6) $$\Delta \left(g_{k}^{\text{obs}},g_{k}^{\text{pred}}\right)=\frac{1}{G}\sum_{k=1}^{G}{\left(\sqrt{g_{k}^{\text{obs}}}-\sqrt{g_{k}^{\text{pred}}}\right)}^{2},$$

i.e., the mean square difference of the square-root transformed frequency distributions. We use a square-root transform to balance the different contributions to Δ. Without this transform large values of *g*_*k *_(i.e., the tips of the U-shaped distribution at *k *= 1 and *k *= *G*) are weighed too heavily; with a logarithm transform small values of *g*_*k *_(i.e., the intermediate part of gene frequency distribution, 2 ≤ *k *≤ *G *- 1) get proportionally too much weight. The fitted model parameters are reported in Table 2, and the corresponding distance Δ in Table 1. See Additional file 3 for Matlab scripts utilized to estimate the best fit parameters for each model.

### Estimating gene uptake rates from transformation frequency

DNA uptake rates are generally presented as transformation frequencies, i.e., the fraction of colony forming units (CFUs) which have taken up a marker sequence relative to the total number of CFUs. Let us denote *ϵ *as the transformation frequency in an uptake experiments in which growing cells are exposed to DNA for a time *T *in exponential growth phase. Consider the division rate of the cells to be *r*, irrespective of whether they have taken up the marker sequence or not. Hence, we can write the dynamics for the population of cells without, *s*(*t*), and with, *m*(*t*), the marker sequence: *ds*/*dt *= *rs-σs *and *dm/dt *= *rm *+ *σs*, where *σ *is the gene uptake rate we would like to estimate. The solutions to these equations are *s*(*t*) = *s*_0_*e*^(*r-*σ)*t *^and *m*(*t*) = *s*_0_*e*^*rt *^(1 - *e*^*-σt*^), where *s*_0 _is the initial population of cells. Hence, at the end of the experiment, *ϵ *= *m*(*T*)/(*s*(*T*) + *m*(*T*)) or ϵ = 1 - *e*^-*σT*^. Hence, *σ *can be estimated from the measurement of *ϵ *by solving $\sigma =-\frac{1}{T}\text{log}\left(1-\varepsilon \right)$.

## Authors' contributions

BH and JSW designed the study, developed the model, analyzed models and data, and co-wrote the manuscript. Both authors read and approved the final manuscript.

## Supplementary Material

Additional file 1 **Appendix**. Composed appendix, including text and figures providing additional information on how to derive gene frequency distributions, pan and core genome scaling, and calculate model fits. Click here for file

Additional file 2 **Gene frequency data**. Raw data of the number of genes found in number of genomes for each of the 6 species analyzed here (in RTF format). Click here for file

Additional file 3 **Model fit scripts**. A set of Matlab files to estimate the best fit parameters for each of the 4 models when given gene frequency data (in RTF format). Click here for file
