# Supplementary material for: A neutral theory of genome evolution and the frequency distribution of genes
Source: BMC Genomics. 2012 May 21;13:196. doi: 10.1186/1471-2164-13-196 (PMC3386021; doi:10.1186/1471-2164-13-196)
Supplement: Additional file 1 — Appendix. Composed appendix, including text and figures providing additional information on how to derive gene frequency distributions, pan and core genome scaling, and calculate model fits. [file 1471-2164-13-196-S1.PDF]

Additional File 1: Appendix
A Neutral Theory of Genome Evolution and the Frequency Distribution of Genes

Bart Haegeman[1,*] and Joshua S. Weitz[2,3,†]

[1] *INRIA Sophia Antipolis — Méditerranée,*
UMR MISTEA, 2 pl. Viala, 34060 Montpellier, France

[2] *School of Biology, Georgia Institute of Technology, Atlanta, GA 30332, USA*

[3] *School of Physics, Georgia Institute of Technology, Atlanta, GA 30332, USA*

(Dated: March 25, 2012)

APPENDIX S1: ANALYSIS OF CONSTANT POPULATION SIZE MODEL

The constant population size model defined in the main text can be analyzed using the standard techniques of population genetics [1, 5]. We are interested in the average gene frequencies g_k, i.e., the expected number of genes that appear in k of the G sampled genomes. First, notice that the average frequencies g_k satisfy

$$g_k = M\widetilde{g}_k, \tag{S1}$$

with $\widetilde{g}_k$ the average gene frequencies at a single locus (recall that in our model the genomes consist of M genes). For a gene to be present in k of the G genomes, it has to have entered the population during a gene transfer event, and consequently, to have spread inside the population due to genetic drift. Recall that reproduction events are described by birth-death events of the Moran model, and that gene transfer events are analogous to mutation events in the infinitely many alleles models. Hence, the gene frequencies distribution at a single locus is governed by the infinitely many alleles version of the Moran model, with reproduction rate ρ per individual and mutation rate $\mu = \sigma/M$ per individual. Using the Moran model results [1, 5], the average gene frequencies $\tilde{g}_k$ are given by

$$\widetilde{g}_k = \frac{\theta}{k}\frac{G!}{(G-k)!}\frac{(\theta)_{G-k}}{(\theta)_G}, \tag{S2}$$

with

$$\theta = \frac{N\mu}{\rho} = \frac{N\sigma}{M\rho}.$$

---

*Electronic address: `bart.haegeman@inria.fr`; URL: `http://bart.haegeman.free.fr`
†Electronic address: `jsweitz@gatech.edu`; URL: `http://ecotheory.biology.gatech.edu`

Typeset by REVTEX

Combining (S1) and (S2) gives

$$g_k = \frac{M\theta}{k}\frac{G!}{(G-k)!}\frac{(\theta)_{G-k}}{(\theta)_G}, \tag{S3}$$

which is Eq. (1) of the main text. Notice that, although the average gene frequencies are the same, our model is more complicated than M independent Moran models. Our model predicts non-trivial correlations between gene frequency distributions at different loci.

Some qualitative features of the distribution (S3) can be obtained as follows. Its behavior for small k is determined by the factor $\frac{1}{k}$, which explains the presence of a peak at $k = 1$ for all values of θ and G. Its behavior for k close to G is determined by the factor $\frac{(\theta)_{G-k}}{(G-k)!}$. This factor is constant for $\theta = 1$, decreasing for $\theta > 1$ and increasing for $\theta < 1$. Hence, a peak at $k = G$ is present for $\theta < 1$, which is more pronounced for smaller θ, and which is steepest for $\theta \to 0$, where it has a $\frac{1}{G-k}$ dependence. In short, the gene frequency distribution is U-shaped with peaks at $k = 1$ and $k = G$ for $\theta < 1$ and for all values of G.

The genericity of the U-shape is illustrated in Figure 1 (for different values of θ) and in Figure S1 (for different sample sizes G). The peaks of the U-shaped distribution are steeper for larger G. The height of the peak at $k = 1$, i.e. the number of genes appearing in a single genome of the sample, increases with θ and is almost independent of G. The height of the peak at $k = G$, i.e. the observed core genome size M_{core} in a sample of G genomes, is given by

$$M_{\text{core}} = g_G = \frac{M\theta}{G}\frac{G!}{(\theta)_G}. \tag{S4}$$

It decreases with θ, decreases with G, and tends to zero as $\frac{1}{G^\theta}$ for $G \to \infty$. The total number of different genes in the sample, i.e. the observed pan genome size M_{pan} in a sample of G genomes, is given by

$$M_{\text{pan}} = \sum_{k=1}^{G} g_k = M\theta\big(\psi(\theta + G) - \psi(\theta)\big). \tag{S5}$$

with ψ the digamma function. The pan genome size increases with θ, increases with G, and diverges as $\ln G$ for $G \to \infty$.

The predicted genomic fluidity φ can be obtained as follows. According to Eq. (4) we have to compute the number of unique genes U_k and U_ℓ in a pair of genomes. As we assumed that all genomes have the same number of genes M, we have $M_k = M_\ell = M$ and also $U_k = U_\ell$. The number of unique genes can be obtained by taking a subsample of size 2 from the sample of $G > 2$ genomes. Alternatively, we can apply Eq. (1) directly for $G = 2$. The average number of unique

genes is

$$\langle U_k + U_\ell \rangle = g_1 = 2M\theta \frac{\theta}{(\theta)_2} = \frac{2M\theta}{\theta + 1}.$$

Substituting this into (4), we obtain Eq. (5) of the main text.

APPENDIX S2: FURTHER DATA COMPARISON FOR CONSTANT POPULATION SIZE MODEL

In Figure S2 we plot the gene frequency distributions on a double logarithmic scale. This allows us to zoom in on the distribution g_k for small k. With this scaling of the axes, power-law dependence is represented as a straight line. The distributions predicted by model A (red line) have a power-law dependence $g_k \sim k^{-\gamma}$ with exponent $\gamma = 1$. The empirical gene frequency distributions (black circles) are close to a power law with exponent $\gamma \approx 2$.

The predictions for the observed core genome size M_{core} and the observed pan genome size M_{pan} are compared to the data in Figure S3. There is a reasonable agreement between model and data, although our model is systematically underestimating the core and pan genome sizes. Recall that the fits in Figure 3 have a similar deviation: the peaks of the U-shaped gene frequency distribution are also systematically underestimated. The predictions for core and pan genome could be improved by using a distance function different from (6), e.g.

$$\Delta_{\text{lin}}\left(g_k^{\text{obs}}, g_k^{\text{pred}}\right) = \frac{1}{G} \sum_{k=1}^{G} \left(g_k^{\text{obs}} - g_k^{\text{pred}}\right)^2. \tag{S6}$$

This distance function would attribute more weight to the tips of the distribution at $k = 1$ and $k = G$. However, this would lead to a worse fit for the intermediate part of the gene frequency distribution (for $2 \leq k \leq G - 1$). We caution however that extrapolating the real pan genome size in the population from the observed pan genome size in a sample can lead to very different results and should be avoided altogether, as we have argued previously [4].

APPENDIX S3: ANALYSIS OF VARIABLE POPULATION SIZE MODEL

To define the model with variable population size, we use the equivalence of the Moran and Wright-Fisher models of population genetics [1, 5]. The equivalence is valid for large population size N, a condition that is satisfied for bacterial populations. In a birth-death event of the Wright-Fisher model the entire population is updated simultaneously; each individual in generation t

chooses its parent from the previous generation. This allows us to impose a different population size $N(t)$ at each time step t. The birth-death events of the Moran model are less flexible in this respect. However, due to the model equivalence, we can extend the Moran-like model for constant population size with a Wright-Fisher-like model for variable population size.

We are interested in the average gene frequencies g_k. As for the constant population size model, it suffices to compute the average gene frequencies $\widetilde{g}_k$ at a single locus, and apply (S1) to get the average gene frequencies g_k for all genes in the genomes. The variable population size model can be analyzed with coalescence theory [2]. The coalescence process describes when ancestral lineages of the sampled individuals started diverging in the past. Superimposing the events that cause the genetic divergence (mutation events in population genetics, and gene transfer events in our model) allows us to analyze different models. The integration of the coalescence process and the mutation process is particularly simple for the infinitely many alleles models, because lineages in which the mutation process introduces a new allele can be discarded from the coalescent process.

To describe the coalescence process for a large and variable population size $N(t)$, we rescale time with the coalescence time scale

$$\text{coalescence time scale} = \frac{N_0}{2\rho}$$

with $N_0 = N(t_0)$ the population size at the present time t_0 and ρ the reproduction rate per individual. If there are q ancestral lineages at a time t in the past, then the next coalescence event (looking backward in time) happens with a rate

$$\text{coalescence rate} = \frac{q(q-1)}{2}\frac{N_0}{N(t)} = \frac{q(q-1)}{2\lambda(t)},$$

which is time-dependent. To superimpose the gene transfer events on this coalescence process, we express the gene transfer rate $\mu = \sigma/M$ per individual per locus in the rescaled time,

$$\text{gene transfer rate} = \frac{N_0}{2\rho}\mu = \frac{N_0}{2\rho}\frac{\sigma}{M} = \frac{\theta_0}{2}.$$

Similarly, for an exponentially growing population (2), we have to express the growth rate α in the rescaled time to get the dimensionless parameter β, see Eq. (3).

To compute the single-locus average gene frequencies $\widetilde{g}_k$, we introduce the probabilities $\widetilde{p}_k$ that a gene is present in a given subset of k genomes and absent in the other $G-k$ genomes. We have

$$\widetilde{g}_k = \frac{G!}{k!(G-k)!}\widetilde{p}_k. \tag{S7}$$

The probabilities $\widetilde{p}_k$ can be computed by summing the probabilities of all contributing realizations of the coalescence with gene transfer process. For a realization to contribute, all ancestral lineages

of the k genomes should coalesce together before one of them undergoes a gene transfer event (in the locus we are considering). Moreover, all ancestral lineages of the $G - k$ genomes should undergo a gene transfer event before one of them coalesce with an ancestral lineage of the k genomes. The summation of all these probabilities can be carried out by integrating a system of ordinary differential equations (ODEs), running backward in time, from the present to the common ancestor of the G sampled genomes. The ODE system keeps track of the number of the q ancestral lineages in the coalescent process, distinguishing three types of lineages:

- A lineage of type A has descendants in the subset of k genomes;
- A lineage of type B has no descendants in the subset of k genomes and has not undergone gene transfer events;
- A lineage of type C has no descendants in the subset of k genomes and has undergone gene transfer events.

The variables of the ODE system are the probabilities $\widetilde{p}_k(q_{\mathrm{A}}, q_{\mathrm{B}}, q_{\mathrm{C}}; t)$ to have q_{A} lineages of type A, q_{B} lineages of type B and q_{C} lineages of type C at the rescaled time t. The dynamics of these variables are governed by the following transitions:

- Gene transfer events in type A lineages occur with rate $q_{\mathrm{A}}\frac{\theta_0}{2}$. If there are still type A lineages to coalesce (i.e., $q_{\mathrm{A}} > 1$), then the realization should be discarded.
- Gene transfer events in type B lineages occur with rate $q_{\mathrm{B}}\frac{\theta_0}{2}$. The number q_{B} should be decreased by one, and the number q_{C} should be increased by one.
- Gene transfer events in type C lineages occur with rate $q_{\mathrm{C}}\frac{\theta_0}{2}$. The configuration $(q_{\mathrm{A}}, q_{\mathrm{B}}, q_{\mathrm{C}})$ remains unchanged.
- Coalescence events between two type A lineages occur with rate $\frac{q_{\mathrm{A}}(q_{\mathrm{A}}-1)}{2\lambda(t)}$. The number q_{A} should be decreased by one.
- Coalescence events between two type B lineages occur with rate $\frac{q_{\mathrm{B}}(q_{\mathrm{B}}-1)}{2\lambda(t)}$. The number q_{B} should be decreased by one.
- Coalescence events between two type C lineages occur with rate $\frac{q_{\mathrm{C}}(q_{\mathrm{C}}-1)}{2\lambda(t)}$. The number q_{C} should be decreased by one.

- Coalescence events between a type A lineage and a type B lineage occur with rate $\frac{q_A q_B}{\lambda(t)}$. The realization should be discarded.

- Coalescence events between a type A lineage and a type C lineage occur with rate $\frac{q_A q_C}{\lambda(t)}$. The number q_C should be decreased by one.

- Coalescence events between a type B lineage and a type C lineage occur with rate $\frac{q_B q_C}{\lambda(t)}$. The number q_C should be decreased by one.

The initial condition for the ODE system at the present time t_0 is

$$\widetilde{p}_k(q_A, q_B, q_C; t_0) = \begin{cases} 1 & \text{if } q_A = k,\ q_B = G - k,\ q_C = 0 \\ 0 & \text{otherwise.} \end{cases}$$

For large time t (looking backward in time), all lineages have coalesced and only $\widetilde{p}_k(1, 0, 0; t)$ is different from zero,

$$\widetilde{p}_k = \lim_{t \to -\infty} \widetilde{p}_k(1, 0, 0; t). \tag{S8}$$

Substituting this into (S1) and (S7) we obtain the gene frequencies g_k. We have checked that this algorithm gives the correct result (1) for the case of constant population size, and that it agrees well with simulation results for the case of variable population size.

APPENDIX S4: FURTHER DATA COMPARISON FOR VARIABLE POPULATION SIZE MODEL

Figure S5 shows the distance between an empirical gene frequency distribution and the theoretical gene frequency distribution as a function of the parameters θ_0 and β. The fitting error has a valley-like structure (the valley is represented in brownish colors in Figure S5). This means that parameter combinations inside the valley have a similar, small fitting error, whereas parameter combinations on the hills of the valley have a larger fitting error. Hence, the optimal parameters, i.e., those with minimal fitting error, are necessarily located inside the valley, but their exact location inside the valley might be difficult to determine, and depend on the details of the fitting procedure [3]. For example, the optimum can shift inside the valley when using distance function (S6) instead of (6). Note that the fitted parameters for model A ($\theta = 0.40$ for the *E. coli* data used in Figure S5) and model B ($\theta_0 = 2.1, \beta = 17$ for the *E. coli* data) correspond to values of the

genomic fluidity equal to the empirical genomic fluidity (i.e., parameter combinations on the thick white line in Figure S5). This is also valid for the other analyzed groups of genomes, see Table 1.

The fitted gene frequency distributions are plotted on a double logarithmic scale in Figure S2. The distributions predicted by model B (blue line) are well approximated by a power law $g_k \sim k^{-\gamma}$ for small k. The decrease at small k is steeper than for model A (the exponent is larger, close to $\gamma \approx 2$), and corresponds well with the data.

APPENDIX S5: ANALYSIS OF MODELS WITH EXPLICIT CORE GENOME

We compute the average gene frequencies g_k for models C and D described in the main text. First, note that model C is a special case of model D, in which the gene transfer parameter θ_2 of the least fluid part of the genome has been set to zero. Hence, it suffices to analyze model D. As we are interested in the average gene frequencies, we can consider each locus separately. There are $\lambda_1 M$ loci with replacement rate σ_1, and $\lambda_2 M$ loci with replacement rate σ_2. Hence, there are $\lambda_1 M$ loci with gene frequencies (1) and $\theta = \theta_1 = \frac{N\sigma_1}{M\rho}$, and $\lambda_2 M$ loci with gene frequencies (1) and $\theta = \theta_2 = \frac{N\sigma_2}{M\rho}$. As a result, the average gene frequencies g_k per genome for model D are

$$g_k = \lambda_1 \frac{M\theta_1}{k} \frac{G!}{(G-k)!} \frac{(\theta_1)_{G-k}}{(\theta_1)_G} + \lambda_2 \frac{M\theta_2}{k} \frac{G!}{(G-k)!} \frac{(\theta_2)_{G-k}}{(\theta_2)_G}. \tag{S9}$$

Putting $\theta_2 = 0$, we find the average gene frequencies g_k per genome for model C,

$$g_k = \begin{cases} \lambda_1 \dfrac{M\theta_1}{k} \dfrac{G!}{(G-k)!} \dfrac{(\theta_1)_{G-k}}{(\theta_1)_G} & \text{if } k < G \\ \lambda_1 \dfrac{M\theta_1}{G} \dfrac{G!}{(\theta_1)_G} + \lambda_2 M & \text{if } k = G. \end{cases} \tag{S10}$$

APPENDIX S6: FURTHER DATA COMPARISON FOR MODELS WITH EXPLICIT CORE GENOME

We comment on the behavior of the fitted gene frequency distributions g_k for small k, see Figure S2. The solution of model C (yellow line) has the same behavior as the solution of model A, resulting in a poor fit with the data. In model D the part of the genome with large fluidity (large θ) determines the left part of the distribution g_k. For large θ the simple power-law approximation $g_k \sim \frac{1}{k}$ is only accurate for $k \ll 1$. It should be replaced by the approximation $g_k \sim \frac{a^k}{k}$ with $a < 1$ for $k = 1, 2, \ldots$. The resulting distribution g_k (green line) decreases faster than $\frac{1}{k}$, and the fit with the empirical data is good.

From (S9) we can obtain a prediction for the observed core genome size $M_{\rm core}$,

$$M_{\rm core} = g_G = \lambda_1 \frac{M\theta_1}{G} \frac{G!}{(\theta_1)_G} + \lambda_2 \frac{M\theta_2}{G} \frac{G!}{(\theta_2)_G}, \tag{S11}$$

and a prediction for the observed pan genome size $M_{\rm pan}$,

$$M_{\rm pan} = \sum_{k=1}^{G} g_k = \lambda_1 M\theta_1 \big(\psi(\theta_1 + G) - \psi(\theta_1)\big) + \lambda_2 M\theta_2 \big(\psi(\theta_2 + G) - \psi(\theta_2)\big). \tag{S12}$$

These predictions are compared to the data in Figure 6. The three-parameter model D agrees much better with the data than the one-parameter model A, see Figure S3. However, despite the excellent fit, we emphasize that extrapolating the real pan genome size in the population from the observed pan genome size in a sample can lead to very different results and should be avoided altogether, as we have argued previously [4].

---

[1] W. Ewens. *Mathematical Population Genetics.* Springer, New York, 2nd edition, 2005.

[2] R. C. Griffiths and S. Tavaré. Sampling theory for neutral alleles in a varying environment. *Philos Trans R Soc Lond B Biol Sci*, 344(1310):403–410, 1994.

[3] R. N. Gutenkunst, J. J. Waterfall, F. P. Casey, K. S. Brown, C. R. Myers, and J. P. Sethna. Universally sloppy parameter sensitivities in systems biology models. *PLoS Comput Biol*, 3(10):e189, 2007.

[4] A. O. Kislyuk, B. Haegeman, N. Bergman, and J. S. Weitz. Genomic fluidity: an integrative view of gene diversity within microbial populations. *BMC Genomics*, 12(1):32, 2011.

[5] J. Wakeley. *Coalescent Theory: An Introduction.* Roberts and Company, Greenwood Village, 2009.

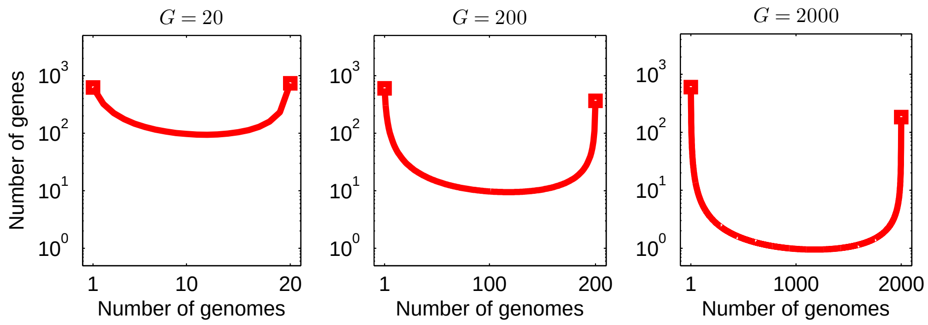

FIG. S1: Effect of sample size on gene frequency distribution. Distributions predicted by model A are shown as a function of the number of sampled genomes G. The number of genes appearing in a single genome and the number of genes appearing in all genomes (that is, the core genome size) are indicated by squares. Genome size $M = 2000$ and gene transfer parameter $\theta = 0.3$. Sample size: in left panel, $G = 20$; in middle panel $G = 200$; in right panel $G = 2000$.

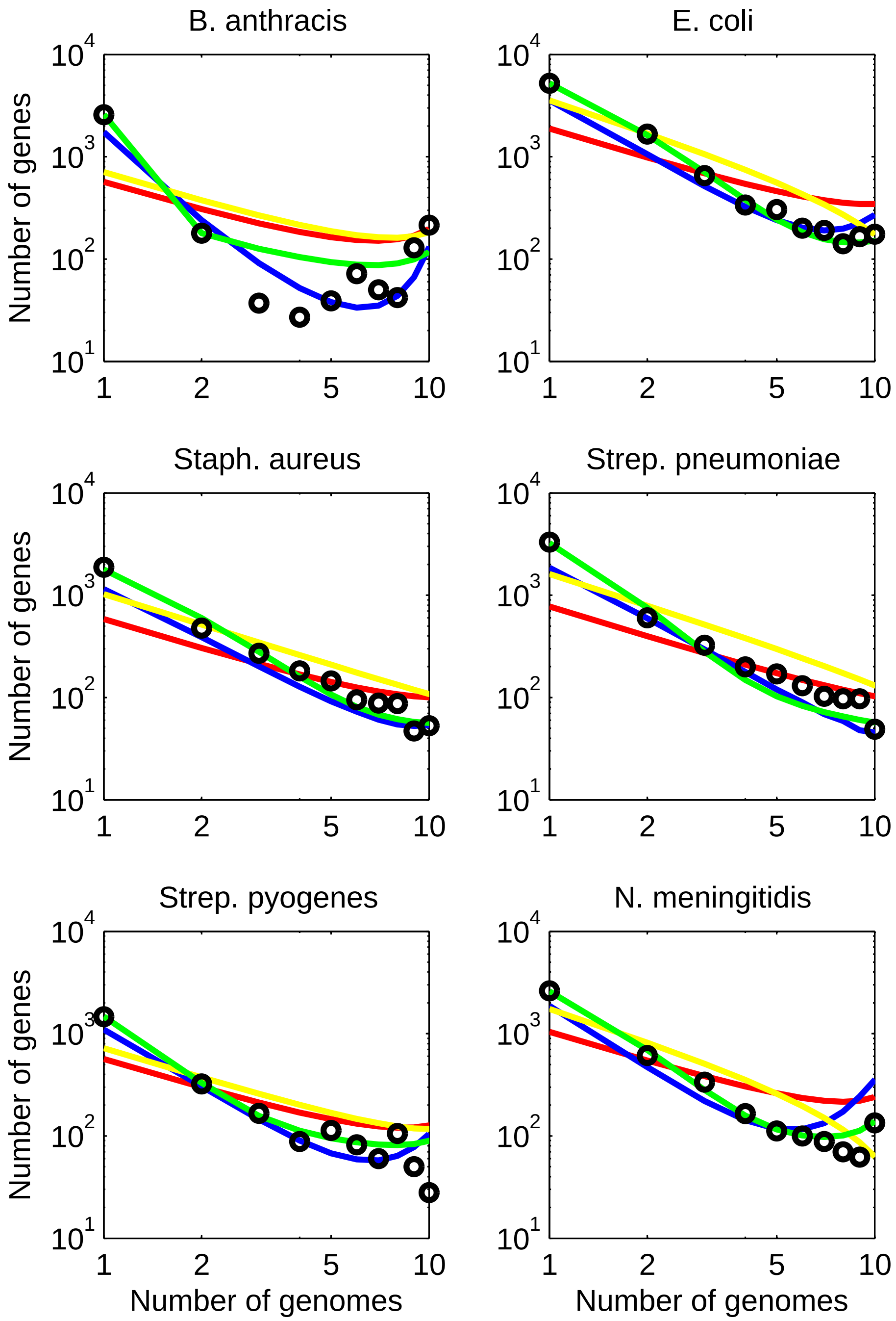

FIG. S2: Power-law behavior of gene frequency distributions for small number of genomes. We compare the predictions of the four models (model A in red, model B in blue, model C in yellow and model D in green) with the empirical gene frequency distributions (black circles). Power-law dependence at small number of genomes is represented as a straight line on the double logarithmic scale. Note that the total number of genomes is larger than the number of genomes shown here (so that the U-shape cannot always be seen in this plot).

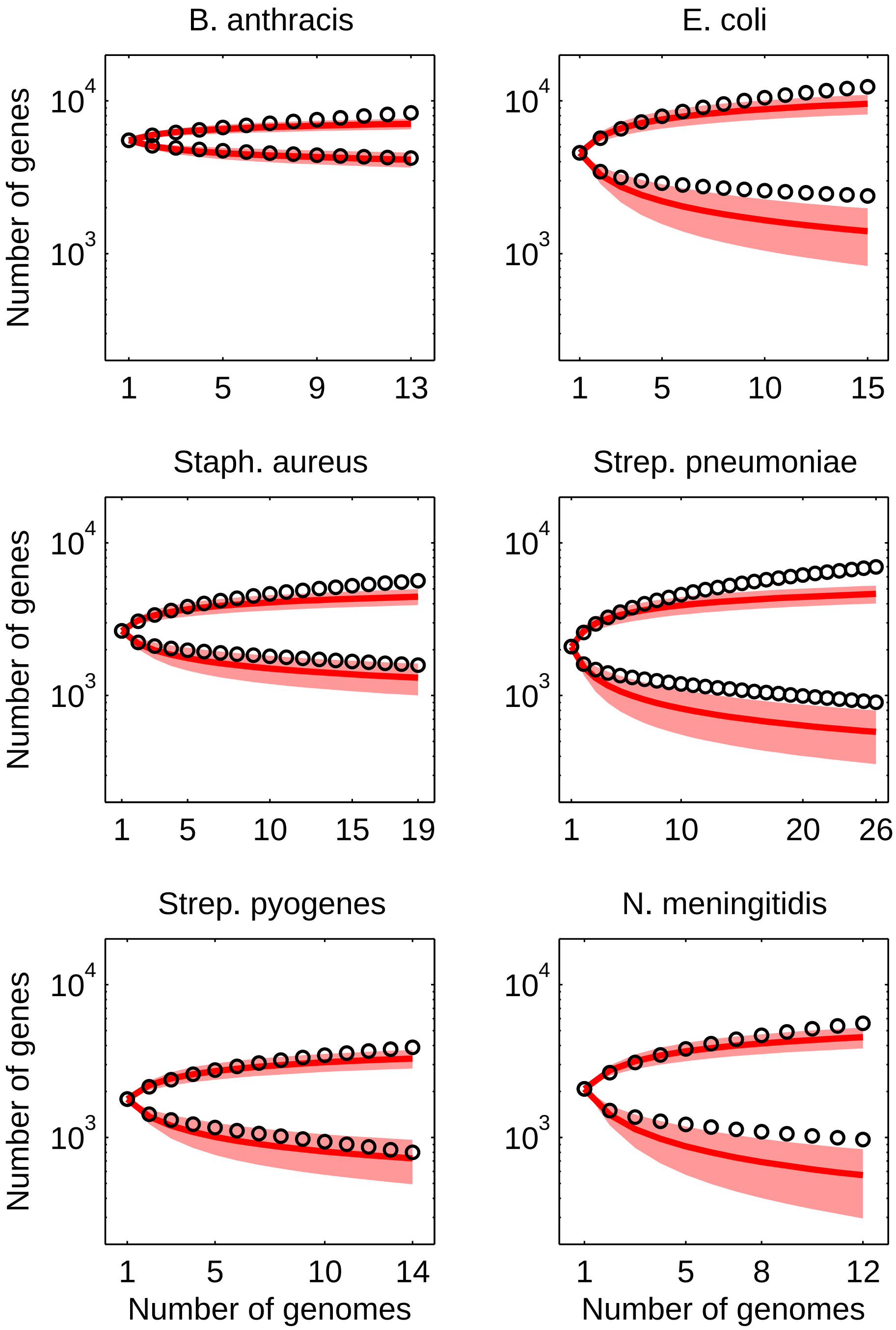

FIG. S3: Predictions for observed core and pan genome size for Model A. We used the gene transfer parameter θ obtained from fitting the gene frequency distribution (see Figure 3) to evaluate the predicted core genome size (S4) and the predicted pan genome size (S5). Black circles: data; red line: mean prediction; red shaded region: standard deviation of prediction. The increasing curves are for the pan genome; the decreasing curves are for the core genome.

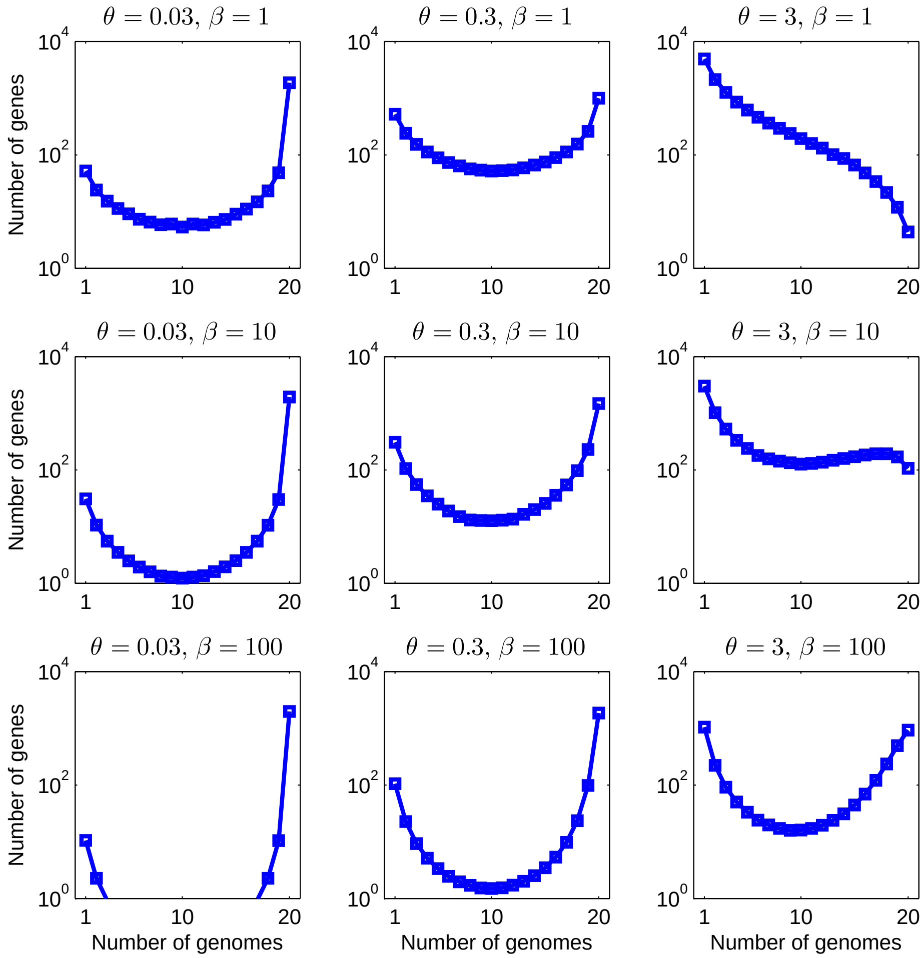

FIG. S4: Gene frequency distributions for model B. Genome size $M = 2000$ and sample size $G = 20$. Gene transfer parameter θ_0: in left column, $\theta_0 = 0.03$; in middle column, $\theta_0 = 0.3$; in right column: $\theta_0 = 3$. Population growth parameter β: in upper row, $\beta = 1$; in middle row, $\beta = 10$; in lower row, $\beta = 100$.

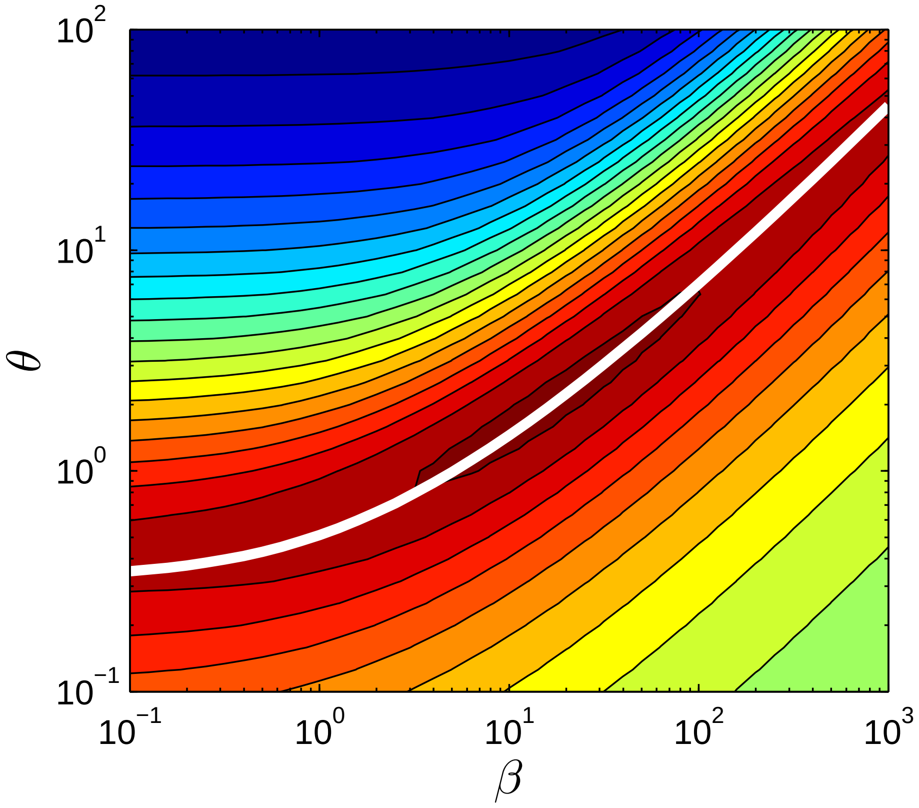

FIG. S5: Fitting error for model B. The distance Δ between the observed gene frequency distribution for *E. coli* and the predicted gene frequency distribution for model B is plotted as a function of the model parameters θ_0 and β. Warm colors correspond to small errors; cold colors correspond to large errors. The level lines, indicated by thin black lines, vary from $\Delta = 70$ to $\Delta = 2400$ in regular steps. The parameter combinations on the thick white line have the same genomic fluidity $\varphi = 0.25$, equal to the genomic fluidity estimated from the data.
