# Supplementary material for: A neutral theory of genome evolution and the frequency distribution of genes
Source: BMC Genomics. 2012 May 21;13:196. doi: 10.1186/1471-2164-13-196 (PMC3386021; doi:10.1186/1471-2164-13-196)
Supplement: Additional file 2 — Gene frequency data. Raw data of the number of genes found in number of genomes for each of the 6 species analyzed here (in RTF format). [file 1471-2164-13-196-S2.RTF]

Bacillus anthracis, 2578,179,37,27,39,72,50,42,129,215,181,596,4221Escherichia coli, 5234,1668,656,337,305,201,190,141,165,175,89,142,184,527,2392Neisseria meningitidis, 2628,611,336,165,112,100,88,70,62,134,328,967Staphylococcus aureus, 1872,476,270,182,145,95,88,87,47,53,37,48,39,35,29,63,84,418,1583Streptococcus pneumoniae, 3308,603,324,199,170,130,103,97,97,49,49,46,42,41,42,30,33,28,23,45,22,38,49,123,364,904Streptococcus pyogenes, 1466,322,166,88,113,82,60,106,50,28,40,109,467,798
