# Supplementary material for: A neutral theory of genome evolution and the frequency distribution of genes
Source: BMC Genomics. 2012 May 21;13:196. doi: 10.1186/1471-2164-13-196 (PMC3386021; doi:10.1186/1471-2164-13-196)
Supplement: Additional file 3 — Model fit scripts. A set of Matlab files to estimate the best fit parameters for each of the 4 models when given gene frequency data (in RTF format). [file 1471-2164-13-196-S3.RTF]

Supplementary File 2 - Matlab scriptsREADMEThis rtf files contains the analysis files used in [1].  Questions and comments can be sent to Joshua Weitz <jsweitz@gatech.edu>.[1] Haegeman B, Weitz JS (2011) A neutral theory of genome evolution and the frequency distribution of genes. Manuscript.model_A_genefreq.m - Matlab script which calculates the gene frequency distribution under model A (constant population size).  It takes as inputs - the number of genomes N, - the number of genes per genome M, - the gene transfer parameter theta.model_B_genefreq.m - Matlab script which calculates the gene frequency distribution under model B (exponentially growing population size). It takes as inputs - the number of genomes N, - the number of genes per genome M, - the gene transfer parameter theta0, - the population growth parameter beta.If the number of genomes N is large (e.g., N=20), this computation can take a considerable amount of time.model_B_genefreq_rhs.m - Auxiliary Matlab script used by model_B_genefreq.m.model_C_genefreq.m - Matlab script which calculates the gene frequency distribution under model C (rigid core genome). It takes as inputs - the number of genomes N, - the number of genes per genome M, - the fraction lambda2 of the rigid core genome, - the gene transfer parameter theta1 of the fluid genome part.model_D_genefreq.m - Matlab script which calculates the gene frequency distribution under model D (flexible core genome). It takes as inputs - the number of genomes N, - the number of genes per genome M, - the fraction lambda2 of the flexible core genome, - the gene transfer parameter theta1 of the most fluid genome part, - the gene transfer parameter of the least fluid genome part.model_error.m - Matlab script which calculates the distance Delta between an empirical gene frequency distribution and a model prediction (see Eq. (6) in [1]). It takes as inputs  - the empirical gene frequency distribution - the number of genomes N, - the number of genes per genome M, - the model (1 for A, 2 for B, 3 for C, 4 for D) - the model parameters.model_fitparams.m - Matlab script which calculates the combination of model parameters that minimizes the distance Delta between an empirical gene frequency and the model prediction. It takes as inputs - the empirical distribution, - the model (1 for A, 2 for B, 3 for C, 4 for D).It returns as outputs - the parameters of the model fit, - the distance between the empirical distribution and the model fit, - the gene frequency distribution of the model fit.
%%% model_A_genefreq.m %%%function genefreq=model_A_genefreq(N,M,theta)% function genefreq=model_A_genefreq(N,M,theta)% N = sample size (number of genomes in sample)% M = genome size (number of genes in genome)% theta = gene transfer parameteraux = 1:N;genefreq = M*theta./aux.* ...    exp(gammaln(N+1)-gammaln(N+1-aux) ...    +gammaln(theta+N-aux)-gammaln(theta+N));end
function popstr=model_B_genefreq(N,M,th,be)%%% model_B_genefreq.m %%%% function popstr=model_B_genefreq(N,M,th,be)% N = sample size (number of genomes in sample)% M = genome size (number of genes in genome)% th = gene transfer parameter% be = population growth parameter% if the number of genomes N is large (e.g., N=20),% computation can take a considerable amount of time.if N==1,    popstr=M;    returnendn=N; popstr=zeros(1,n);for k=1:n,    % construct index vectors    qq=[]; mm=[]; ll=[];    for q=1:n,        for m=max([1 k+q-n]):q,            aux=max([1 k+q-n]):min([k m]);            ll=[ll aux];            mm=[mm m*ones(1,length(aux))];            qq=[qq q*ones(1,length(aux))];        end    end    % construct transitions    m1a=zeros(1,sum(ll>1)); i1a=m1a; j1a=m1a; c1a=0;    m1b=zeros(1,sum((ll==1)&(qq>1))); i1b=m1b; j1b=m1b; c1b=0;    m2=zeros(1,sum(mm>ll)); i2=m2; j2=m2; c2=0;    m3=zeros(1,sum(ll>1)); i3=m3; j3=m3; c3=0;    m4=zeros(1,sum(mm-ll>1)); i4=m4; j4=m4; c4=0;    m5=zeros(1,sum(mm>ll)); i5=m5; j5=m5; c5=0;    m6=zeros(1,sum(qq>mm)); i6=m6; j6=m6; c6=0;    for idx=1:length(ll),        l=ll(idx); m=mm(idx); q=qq(idx);        % TRANSITION 1a: prefactor theta        if l>1,            c1a=c1a+1;            m1a(c1a)=l/2;            i1a(c1a)=idx;            j1a(c1a)=-1;        end        % TRANSITION 1b: prefactor theta        if (l==1)&&(q>1),            c1b=c1b+1;            m1b(c1b)=1/2;            i1b(c1b)=idx;            j1b(c1b)=1;        end        % TRANSITION 2: prefactor theta        if m>l,            c2=c2+1;            m2(c2)=(m-l)/2;            i2(c2)=idx;            j2(c2)=find((ll==l)&(mm==m-1)&(qq==q));        end        % TRANSITION 3: prefactor N(0)/N(t)        if l>1,            c3=c3+1;            m3(c3)=l*(l-1)/2;            i3(c3)=idx;            j3(c3)=find((ll==l-1)&(mm==m-1)&(qq==q-1));        end        % TRANSITION 4: prefactor N(0)/N(t)        if m-l>1,            c4=c4+1;            m4(c4)=(m-l)*(m-l-1)/2;            i4(c4)=idx;            j4(c4)=find((ll==l)&(mm==m-1)&(qq==q-1));        end        % TRANSITION 5: prefactor N(0)/N(t)        if m>l,            c5=c5+1;            m5(c5)=l*(m-l);            i5(c5)=idx;            j5(c5)=-1;        end        % TRANSITION 6: prefactor N(0)/N(t)        if q>m,            c6=c6+1;            m6(c6)=(q-m)*(q+m-1)/2;            i6(c6)=idx;            j6(c6)=find((ll==l)&(mm==m)&(qq==q-1));        end    end    % solve differential equation    y0=zeros(1,length(ll));    y0(end)=1;    tf=40; if be>0, tf=log(1+be*tf)/be; end    [~,y]=ode15s(@(t,x) model_B_genefreq_rhs(t,x,th,be, ...        m1a,i1a,m1b,i1b,m2,i2,j2,m3,i3,j3,m4,i4,j4,m5,i5,m6,i6,j6), ...        [0 .5 1]*tf,y0);    % extract population structure    popstr(k)=exp(gammaln(n+1)-gammaln(k+1)-gammaln(n-k+1))*y(3,1);endpopstr=M*popstr;end
%%% model_B_genefreq_rhs.m %%%function dx = calc_popstr_varsize_analy_rhs(t,x,th,be,m1a,i1a,m1b,i1b,m2,i2,j2,m3,i3,j3,m4,i4,j4,m5,i5,m6,i6,j6)% function dx = calc_popstr_varsize_analy_rhs(t,x,th,be,m1a,i1a,m1b,i1b,m2,i2,j2,m3,i3,j3,m4,i4,j4,m5,i5,m6,i6,j6)dx=zeros(size(x));dx(1)=dx(1)+th*sum(m1b'.*x(i1b));dx(j2)=dx(j2)+th*m2'.*x(i2);dx(j3)=dx(j3)+exp(be*t)*m3'.*x(i3);dx(j4)=dx(j4)+exp(be*t)*m4'.*x(i4);dx(j6)=dx(j6)+exp(be*t)*m6'.*x(i6);dx(i1a)=dx(i1a)-th*m1a'.*x(i1a);dx(i1b)=dx(i1b)-th*m1b'.*x(i1b);dx(i2)=dx(i2)-th*m2'.*x(i2);dx(i3)=dx(i3)-exp(be*t)*m3'.*x(i3);dx(i4)=dx(i4)-exp(be*t)*m4'.*x(i4);dx(i5)=dx(i5)-exp(be*t)*m5'.*x(i5);dx(i6)=dx(i6)-exp(be*t)*m6'.*x(i6);end
%%% model_C_genefreq.m %%%function genefreq=model_C_genefreq(N,M,frac,theta)% function genefreq=model_C_genefreq(N,M,frac,theta)% N = sample size (number of genomes in sample)% M = genome size (number of genes in genome)% frac = fraction of rigid core genome% theta = gene transfer parameteraux = 1:N;genefreq = M*theta./aux.* ...    exp(gammaln(N+1)-gammaln(N+1-aux) ...    +gammaln(theta+N-aux)-gammaln(theta+N));genefreq = (1-frac)*genefreq;genefreq(end) = genefreq(end)+M*frac;end
%%% model_D_genefreq.m %%%function genefreq=model_D_genefreq(N,M,frac,theta1,theta2)% function genefreq=model_D_genefreq(N,M,frac,theta1,theta2)% N = sample size (number of genomes in sample)% M = genome size (number of genes in genome)% frac = fraction of flexible core genome% theta1 = gene transfer parameter for part outside flexible core% theta2 = gene transfer parameter for flexible core genomeaux = 1:N;aux1 = M*theta1./aux.* ...    exp(gammaln(N+1)-gammaln(N+1-aux) ...    +gammaln(theta1+N-aux)-gammaln(theta1+N));aux2 = M*theta2./aux.* ...    exp(gammaln(N+1)-gammaln(N+1-aux) ...    +gammaln(theta2+N-aux)-gammaln(theta2+N));genefreq = (1-frac)*aux1+frac*aux2;end
%%% model_error.m %%%function [err,aux]=model_error(data,N,M,model,pars)% function [err,aux]=model_error(data,N,M,model,pars)% data = (empirical) gene frequency distribution% N = sample size (number of genomes in sample)% M = genome size (number of genes in genome)% if model==1, model A -- constant population size%     pars = theta% if model==2, model B -- exponentially growing population%     pars = [theta beta]% if model==3, model C -- rigid core genome%     pars = [frac theta] (frac = fraction of core genome)% if model==4, model D -- flexible core genome%     pars = [frac theta1 theta2] (frac and theta2 for core genome)if model == 1,    theta = pars;    if theta < 0,        err = Inf;        return    end    aux = model_A_genefreq(N,M,theta);elseif model == 2,    th = pars(1);    be = pars(2);    if th < 0 || be < 0,        err = Inf;        return    end    aux = model_B_genefreq(N,M,th,be);elseif model == 3,    frac = pars(1);    theta = pars(2);    if frac < 0 || frac > 1 || theta < 0,        err = Inf;        return    end    aux = model_C_genefreq(N,M,frac,theta);elseif model == 4,    frac = pars(1);    theta1 = pars(2);    theta2 = pars(3);    if frac < 0 || frac > 1 || ...       theta1 < 0 || theta2 < 0 || theta1 < theta2,        err = Inf;        return    end    aux = model_D_genefreq(N,M,frac,theta1,theta2);else    disp('not an appropriate model code')    returnenderr = sum((sqrt(data)-sqrt(aux)).^2)/N;end
%%% model_fitparams.m %%%function [pars,err,aux]=model_fitparams(data,model)% function [pars,err,aux]=model_fitparams(data,model)% data = (empirical) gene frequency distribution% if model==1, model A -- constant population size%     pars = theta% if model==2, model B -- exponentially growing population%     pars = [theta beta]% if model==3, model C -- rigid core genome%     pars = [frac theta] (frac = fraction of core genome)% if model==4, model D -- flexible core genome%     pars = [frac theta1 theta2] (frac and theta2 for core genome)N = length(data);aux = 1:N;M = abs(sum(data.*aux))/N;normdata = data/M;if model == 1,    pars0 = .5;elseif model == 2,    pars0 = [.5 .5];elseif model == 3,    pars0 = [.5 .5];elseif model == 4,    pars0 = [.5 2 .5];else    disp('not an appropriate model code')    returnendopts = optimset('TolFun',1e-8,'TolX',1e-8, ...    'MaxFunEvals',1e6,'MaxIter',1e6);pars = fminsearch(@(x) model_error( ...    normdata,N,1,model,x),pars0,opts);[err,aux] = model_error(data,N,M,model,pars);end
